# IL-2-Agonist-Induced IFN-γ Exacerbates Systemic Anaphylaxis in Food Allergen-Sensitized Mice

**DOI:** 10.3389/fimmu.2020.596772

**Published:** 2020-12-11

**Authors:** Christopher W.M. Link, Christina N. Rau, Christopher C. Udoye, Mohab Ragab, Rabia Ü. Korkmaz, Sara Comdühr, Ann-Katrin Clauder, Timo Lindemann, Britta Frehse, Katharina Hofmann, Larissa N. Almeida, Yves Laumonnier, Asmaa El Beidaq, Fred D. Finkelman, Rudolf A. Manz

**Affiliations:** ^1^Institute for Systemic Inflammation Research, University of Lübeck, Lübeck, Germany; ^2^Institute of Nutritional Medicine, University of Lübeck, Lübeck, Germany; ^3^Division of Allergy, Immunology and Rheumatology, Department of Internal Medicine, University of Cincinnati College of Medicine and the Division of Immunobiology, Cincinnati Children’s Hospital Medical Center, Cincinnati, OH, United States

**Keywords:** food allergy, IL-2, IFN-γ, anaphylaxis, murine model

## Abstract

Food allergies are common, costly and potentially life-threatening disorders. They are driven by Th2, but inhibited by Th1 reactions. There is also evidence indicating that IL-2 agonist treatment inhibits allergic sensitization through expansion of regulatory T cells. Here, we tested the impact of an IL-2 agonist in a novel model for food allergy to hen´s egg in mice sensitized without artificial adjuvants. Prophylactic IL-2 agonist treatment expanded Treg populations and inhibited allergen-specific sensitization. However, IL-2 agonist treatment of already sensitized mice increased mast cell responses and allergic anaphylaxis upon allergen re-challenge. These effects depended on allergen-specific IgE and were mediated through IFN-γ, as shown by IgE transfer and blockade of IFN-γ with monoclonal antibodies. These results suggest that although shifting the allergic reaction toward a Treg/Th1 response inhibits allergic sensitization, the prototypic Th1 cytokine IFN-γ promotes mast cell activation and allergen-induced anaphylaxis in individuals that are already IgE-sensitized. Hence, while a Th1 response can prevent the development of food allergy, IFN-γ has the ability to exacerbate already established food allergy.

## Introduction

The Th2 cytokine IL-4 promotes class switching to IgE antibodies, which bind to the high affinity IgE receptor FcεRI on mast cells (1–3). Crosslinking of FcεRI-bound IgE by antigen/allergen can induce type-1 hypersensitivity reactions, including those responsible for food allergies (4, 5). Th1 reactions and their prototypic cytokine IFN-γ can counterbalance Th2 responses, which reduces the production of IL-4 and IgE and thereby inhibits allergic sensitization (6, 7). These observations suggested that restoring the Th1/Th2 balance by increasing Th1 responses could inhibit IgE-mediated allergy (8). This concept is generally well accepted, although it is clear that additional T cell subsets, such as regulatory T cells (Tregs), also limit allergic reactions (9).

Allergy to chicken (Gallus gallus) egg is the second most prevalent food allergy in infants and children, after cow’s milk allergy (10). A recent meta-analysis of European studies suggests an overall prevalence of 1.6% in children aged 2.5 years (11). Chicken egg white (EW) contains several allergenic proteins. The dominant allergenic protein in EW is the trypsin inhibitor ovomucoid, which accounts for approximately 11% of EW protein, while ovalbumin (OVA), the most abundant egg allergen, accounts for 55% of EW protein. Two putative allergens have been found in egg yolk (EY), chicken serum albumin α-livetin and yolk glycoprotein 42 (12, 13). α-livetin has also been identified as the major inhalant allergen in bird-to-egg syndrome (14, 15), which links respiratory hypersensitivity to bird antigens to egg allergy (16). Exposure to pet birds, poultry, etc. can lead to respiratory allergies, including asthma, by sensitizing people to airborne avian allergens (17–21). Blood, feathers and droppings can be sources of these aeroallergens (22, 23), including serum albumin α-livetin. Reciprocal cross-inhibition of IgE-binding between extracts of bird feathers and egg yolk has been observed for patients diagnosed with bird to egg syndrome (24). Thus, although inhalation of avian airborne allergens first leads to respiratory hypersensitivity, subsequent ingestion of cross-reacting serum albumin in egg yolk can provoke gastro-intestinal symptoms, including diarrhea and vomiting.

T regulatory cells (Tregs) are important for maintaining oral tolerance and preventing food allergy (25). These cells can suppress the production of type 2 cytokines, B cell Ig isotype switching to IgE and the effector functions of mast cells and basophils (26). Tregs constitutively express high levels of CD25, a required component of the high-affinity receptor for IL-2, which maintains Treg homeostasis and survival in response to IL 2 (27–29). In the absence of CD25, CD122, and CD132 form a lower affinity receptor for this cytokine, which is predominantly expressed by effector CD8+ T cells and natural killer (NK) cells (30, 31). Accordingly, application of low doses of IL-2 is believed to selectively expand Treg populations, and has demonstrated therapeutic potential against some autoimmune disorders in clinical trials (32–35). Interestingly, it was reported that this treatment inhibits allergic symptoms, but does not impair IgE production in a murine model of food allergy to OVA (36).

IL-2 can be complexed with a mAb that blocks its binding to the low affinity form of the IL-2R to increase its selectivity for cells, such as Tregs, that express the high affinity IL-2R. In mice, that can be achieved with the anti-CD122 mAb JES6. Complexing IL-2 with JES6 also increases its *in vivo* half-life and activity (37). Accordingly, IL-2/JES6 is a potent IL-2 agonist and treatment with IL-2/JES6 can suppress some inflammatory diseases (38–41). IL-2/JES6 can expand both “natural Tregs” and “peripheral Tregs”, two distinct Treg subpopulations that are respectively generated in the thymus without exposure to foreign antigens, or formed after antigen contact in the periphery, respectively (42).

In the current study, we physiologically sensitize mice to egg allergens by intra-tracheal (i.t.) inoculation with EW and EY plasma (EYP, the liquid, lipid-containing fraction of EY) without artificial adjuvant. Upon subsequent oral challenge with the same allergens, the sensitized mice developed diarrhea and anaphylaxis, which manifests as hypothermia. Using this model we found that IL-2/JES6 induces an IFN-γ response; while this response inhibits Th2 cytokine and IgE production during the sensitization phase, it reduces the threshold for IgE-mediated mast cell activation in already sensitized mice.

## Materials and Methods

### Mice

Female Balb/c mice were purchased from Charles River Laboratories (Sulzfeld, Germany) and maintained under pathogen-free housing conditions. Animal studies were performed at the animal facilities of Cincinnati Children’s Hospital Medical Center and the University of Lübeck, with approval from the respective authorities.

IL 2/JES6-complex treatment: IL 2/JES6-complex was prepared by mixing multiples of 1 µg recombinant mouse IL 2 (Immunotools) with 5 µg anti-IL 2 mAb (clone JES6-1A12; purified from culture supernatants of the hybridoma), dissolved in 200 µl sterile Dulbecco’s phosphate buffered saline (DPBS). The solution was incubated for 30 min at 37°C in a CO2-incubator. IL 2/JES6-complexes were administered i.p. as described (42).

IFN-γ-neutralizing antibody treatment: 100 µg anti-IFN-γ mAb (clone: XMG1.2; kindly donated by Katrin Luger, DRFZ Berlin.) in 100 µl PBS was intra-peritoneal administered (i.p.) to allergic mice either one, or three times on three consecutive days, together with IL-2/JES6.

### Experimental Food Allergy to Hen’s Egg

Eggs from the local grocery were swabbed for 5 min with 70% ethanol and subsequently irradiated for 2 min with UV light. EW and EY were separated in autoclaved beakers. The EW was transferred to dialysis tubing (MWCO 6.000–8.000 Da), then dialyzed against distilled water for 48 h at 4°C, lyophilized, and stored at 20°C. EY was diluted 1:3 in sterile DPBS and then centrifuged for 10 min at 13,000 x g, 4°C, after which the supernatant, EYP, was collected and stored at 20°C.

Mice were anesthetized by i.p. injection of 200 μl anesthetics (5 mg/ml Ketanest S, 1.5 mg/ml Rompun in DPBS), restrained and sensitized by i.t. application of 40 µl EYP containing 50 µg EW. The procedure was repeated according to the sensitization schedules. For antigen (Ag) challenges, lyophilized EW was dissolved in sterile DPBS to a concentration of 500 mg/ml, then mixed with an equal volume of EYP. To assess diarrhea development, EW plus EYP was supplemented with food dye. Mice were intra-gastrically challenged with 300 µl of this mixture. Body temperature was measured by rectal thermometry (Physitemp). IgE-Transfer Model of Passive Systemic Anaphylaxis

Mice were i.v. injected with 10 µg of IgE-anti-TNP mAb (clone IgEL2a; purified from hybridoma culture supernatants) in 200 µl sterile DPBS. To induce systemic anaphylaxis 24 h after sensitization, mice were challenged by gastric lavage with 20 mg of TNP-BSA. Body temperature was measured by rectal thermometry.

### ELISAs

To determine anti-OVA IgE and IgG1 levels, white Costar^®^ 96-well plates were coated with 10 µg/ml OVA in Tris/saline buffer (pH 7.2). Briefly, wells were washed and subsequently blocked with 2% skim milk solution. Standards (IgE (Serotec) and IgG1 (Sigma-Aldrich)) were serially diluted 1:2, starting at 200 ng/ml or 500 ng/ml, respectively. After incubation with standards and diluted samples, wells were repeatedly washed and subsequently incubated with biotinylated anti-mouse IgE (BD) or anti-mouse IgG1 (Southern Biotech). Wells were then washed and incubated with Streptavidin-HRP (Thermo Scientific). Following further washing, substrate (Thermo Scientific) was added to the wells and responses were immediately measured with a Luminometer (FlUOstar Omega, BMG Labtech). For quantification of serum MMCP-1, serum was obtained 4 h after oral gavage (o.g.) challenge. MMCP-1 concentrations were determined by ELISA following the manufacturer’s instructions (eBiosciences).

### *In Vivo* Cytokine Capture Assay

IVCCA was performed as described (43). Briefly, mice were i.v. injected with 10 µg of biotinylated anti-mouse IL-4 (clone BVD4-1D11) either alone or in combination with 10 µg of biotinylated anti-mouse IL-13 (clone SZ45-54D1) 4 h before o.g. challenge. Blood was sampled 4 h after o.g. challenge. Serum IL 4 and IL 13 levels were measured by luminescence ELISA.

### ILC Isolation

Mice were sacrificed and the small intestine was extracted, flushed with ice cold 1×Hank’s balanced salt solution (without Ca^2+^ and Mg^2+^; HBSS (w/o)) and patches were removed. Lamina propria lymphocytes were isolated from the entire small intestine using the isolation kit from Miltenyi Biotech (#130-097-410) according to the manufacturer’s manual with slight modifications.

### Flow Cytometry

Single-cell suspensions were prepared from spleen, mesenteric LN and mediastinal LN. Subsequently, they were filtered through a 70-µm cell strainer, washed, and resuspended in PBS/BSA containing anti-FcγRIIB/RIII mAb (clone 2.4G2) for 5 min. eF450-labeled anti-CD4 (GK1.5; eBioscience) and Brilliant Violet 421-labeled anti-CD3 (145-2C11; Biolegend) mAbs were used for surface staining.

For analysis of ILC, lamina propria lymphocytes were isolated as described above and stained for: CD3, (clone 17A2; Biolegend) B220 (clone RA3-6B2; Biolegend), CD11b (clone M1/70; Biolegend), Gr-1 (clone RB6-8C5; Biolegend), Ter-119 (clone TER-119; Biolegend), CD49b (clone DX5; Biolegend), CD326 (clone G8.8; Biolegend), CD25 (clone 3C7; Biolegend), CD127 (clone A7R34; Biolegend) Sca-1 (clone D7; Biolegend) and CD117/c-kit (2B8; BD).

For analysis of intracellular cytokine expression, cells were re-stimulated by PMA/ionomycin and subsequently protein secretion was inhibited by Brefeldin A. Then cells were stained for lineage markers as described above, washed once with PBS, fixed with fixation solution (BioLegend) according to the manufacturer’s protocol and permeabilized with the FoxP3 Perm buffer (BioLegend), for 15 min at RT. Subsequently, cells were washed with ice-cold PBS and intra cellular cytokines IL-10 and IFN-γ were stained by incubation with BV605 labeled anti-IL-10 mAb (clone: JES5-16E3, Biolegend) and APC conjugated anti-IFN-γ mAb (XMG1.2, Biolegend) for 60 min at RT.

### Chloroacetate Esterase (CAE) Staining

Paraffin slides of formaldehyde-fixed jejunum and duodenum were de-paraffinized in a Rotihistol (Roth) bath (3 changes, 10 min. per change) followed by ethanol (Chemsolut) baths of decreasing concentrations (100%, 95%, 70% V/V). De-paraffinized slides were rinsed with phosphate buffer (0.1M, pH 7.6). Washed slides were stained with freshly prepared solutions. First, a new fuchsin solution (40 mg/ml in 2N HCl; Sigma) was mixed with 4% sodium nitrite (Sigma). Meanwhile, naphtol AS-D chloroacetate (2 mg/ml in N,N dimethylformamid; Sigma both) was mixed with phosphate buffer (0.1M, pH 7.6). The new fuchsin-sodium nitrite solution was combined with the naphtol AS-D chloroacetate solution, added dropwise to the slides and incubated for 25 min. The stained slides were washed with distilled water, counterstained with hematoxylin gill´s no. 2 (Sigma) and washed with warm tap water. Slides were dehydrated in an inverse order from the de-paraffination and sealed under coverslips with Vectamount (Vector).

### Statistical Analysis

Data analysis was performed using GraphPad PRISM 6 software, statistical tests are indicated in individual figure legends. A p-value <0.05 was considered significant.

## Results

### Prophylactic IL-2/JES6-Treatment Prevents IgE Production and Sensitization to Hen’s Egg

**Figure 1:**
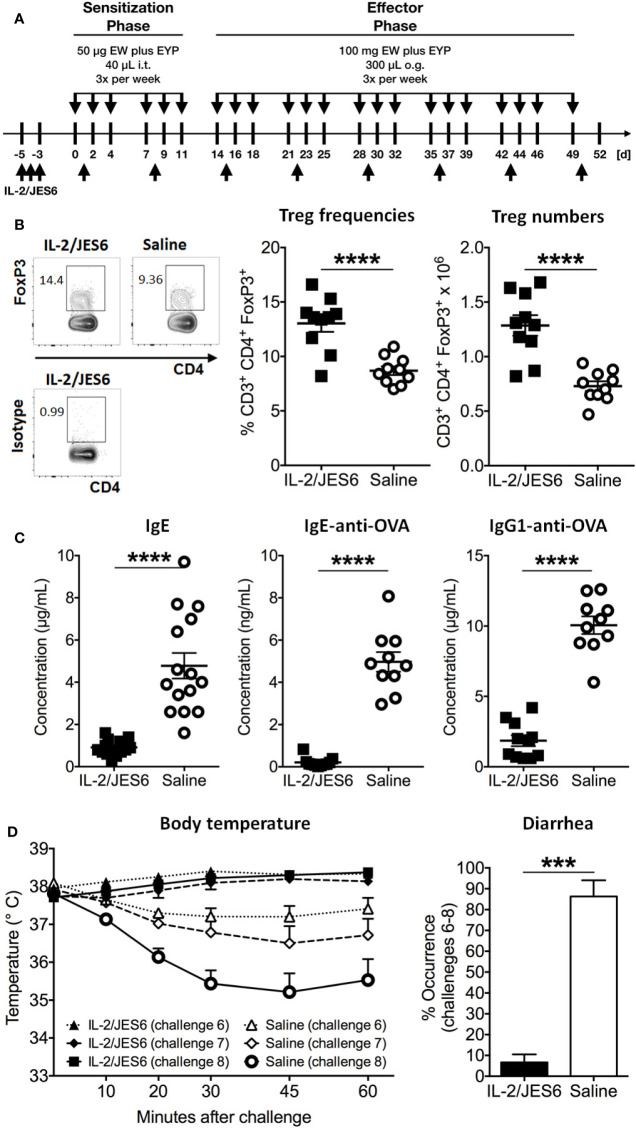
Prophylactic IL-2/JES6 treatment. **(A)** Experimental scheme. Mice were treated with IL 2/JES6 or saline (up-arrows) and sensitized by i.t. inoculations with egg white (EW) plus egg yolk plasma (EYP), as indicated (down-arrows). Later, mice were repeatedly challenged with EW plus EYP. **(B)** CD3+ CD4+ FoxP3+ Treg populations in spleen after the 12th challenge, analyzed by flow cytometry. **(C)** Total serum IgE, ovalbumin (OVA)-specific IgE and IgG1 after six i.t. sensitizations, as assessed by ELISA. **(D)** Rectal temperature curve (left) and diarrhea development (right) after oral antigen challenges six to eight. Data shown from one of two independent experiments (n = 14 15). Data depicted represent mean ± SEM. ***p < 0.001, ****p < 0.0001 (Student’s t-test).

Hen’s egg allergy was induced by i.t. sensitization and oral challenge with EW plus EYP. Before sensitization, Treg populations were expanded by three injections of the IL-2 agonist IL 2/JES6 (**Figure 1A**). Treatment with IL 2/JES6 increased percentages and numbers of CD3+ CD4+ FoxP3+ Tregs in spleen, blood and mesenteric lymph nodes approximately 1.5-fold (**Figure 1B**; **Figure S1**). After sensitization, blood samples were collected to evaluate immune sensitization to hen’s egg. Total IgE and OVA-specific serum IgE and IgG1 levels were all approximately 5-fold reduced in mice treated with IL 2/JES6 (**Figure 1C**). Control mice that were sensitized to and subsequently challenged with hen’s egg developed anaphylaxis (manifested as hypothermia) as early as the sixth oral challenge with EW plus EYP; hypothermia plateaued at a decrease of ~3.0°C by the eighth challenge (**Figure 1D**). 83% of these mice also developed allergic diarrhea subsequent to antigen challenge six. In contrast, mice treated with IL 2/JES6 were completely protected from allergic reactions, with no hypothermia and development of diarrhea in only 6.6% (**Figure 1D**).

In order to investigate possible effects of IL-2/JES6 treatment on sensitivity to histamine, mice were prophylactic treated with IL-2/JES6, subsequently challenged with allergen and finally injected with histamine. Compared to a control group treated with saline, IL-2/JES6 treatment had no effect or slightly increased histamine-induced hypothermia (**Figure S2**). Hence, preventive treatment with the IL-2 agonist IL-2/JES6 blocked anaphylaxis in our model, most likely by expanding Treg populations and decreasing allergen-specific IgE production, rather than by inhibiting the response to histamine. These findings are in accordance with previous findings obtained in murine food allergy to milk proteins (44).

### After Sensitization, IL-2/JES6-Treatment Increases Mast Cell Responsiveness and Exacerbates Acute Allergic Symptoms

**Figure 2:**
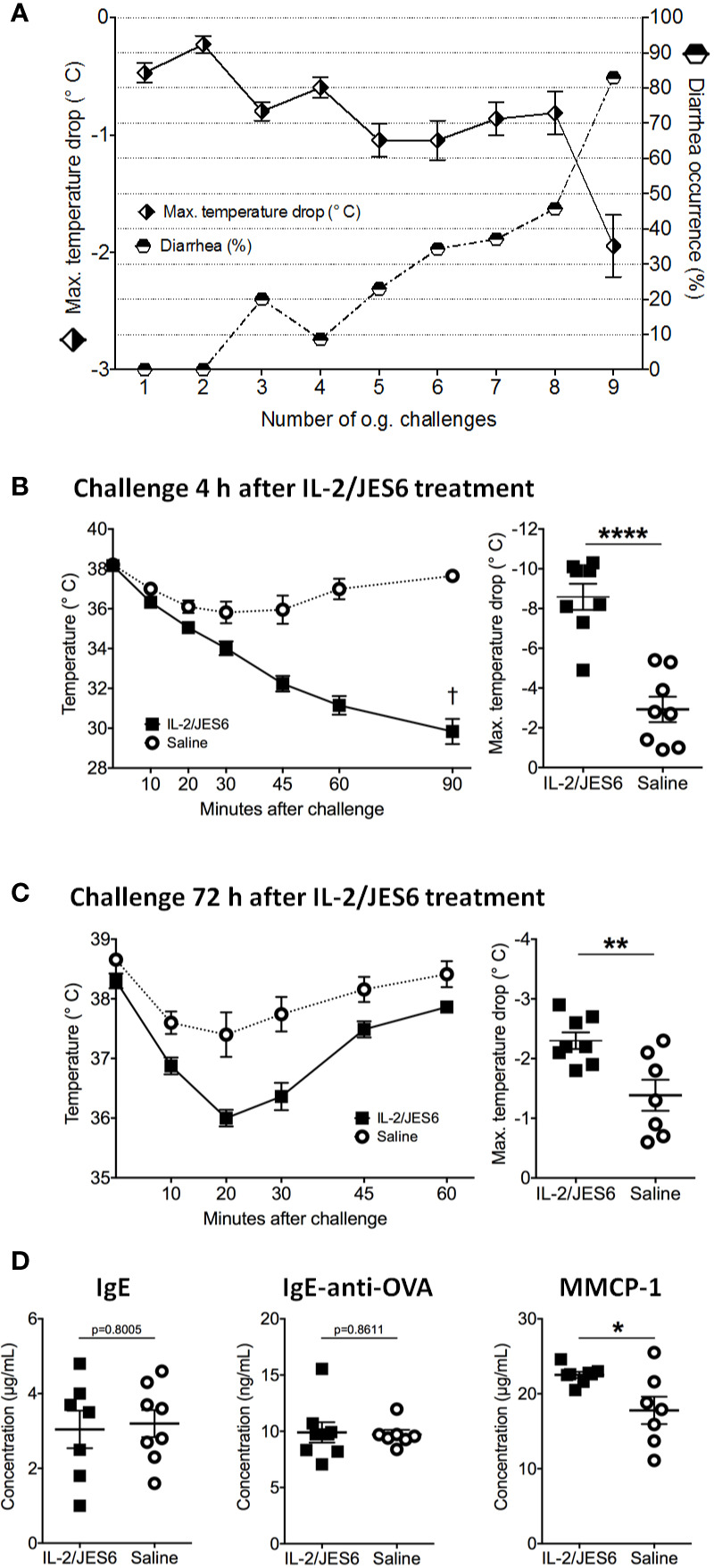
Therapeutic IL-2/JES6 treatment. Sensitized and repeatedly challenged mice were treated with IL-2/JES6 either four or 72 h before the final allergen challenge. **(A)** Hypothermia and diarrhea following allergen challenges, before IL-2/JES6 administration. **(B)** Rectal temperature curve (left) and maximal temperature drop (right) of mice treated with IL-2/JES6 4 h before allergen challenge. **(C)** Rectal temperature curve (left) and maximal temperature drop (right) of mice treated with IL-2/JES6 at 72 h before allergen challenge. **(D)** Total IgE, ovalbumin (OVA)-specific IgE and IgG1 and MMCP-1 in serum measured by ELISA (after challenge 13, 72 h after the last IL-2/JES6 treatment). Data depicted represent mean ± SEM (A: n = 35, **(B–D)** n = 7 8). *p < 0.05, **p < 0.01, ****p < 0.0001 (Student’s t-test).

In order to test IL 2/JES6 in a therapeutic rather than a prophylactic setting, the IL-2 agonist was applied to mice that were already allergic. Repeated challenges with EW plus EYP induced an allergic response in sensitized mice, as manifested by hypothermia and diarrhea (**Figure 2A**). Starting one day after the 11th challenge, allergic mice were treated with IL 2/JES6 on three consecutive days. Four hours after the last IL 2/JES6 treatment, mice received a single oral allergen challenge. Strikingly, all IL 2/JES6-treated mice challenged with allergen developed severe hypothermia and lethal anaphylaxis while the body temperature of control mice decreased much less, comparable to previous challenges (**Figure 2B**). IL 2/JES6 treatment did not alter the frequencies of innate lymphoid cells (ILC) in the small intestine of allergic mice (**Figure S3**), indicating that IL 2/JES6 may have no immediate effect on intestinal IL2C.

**Figure 3:**
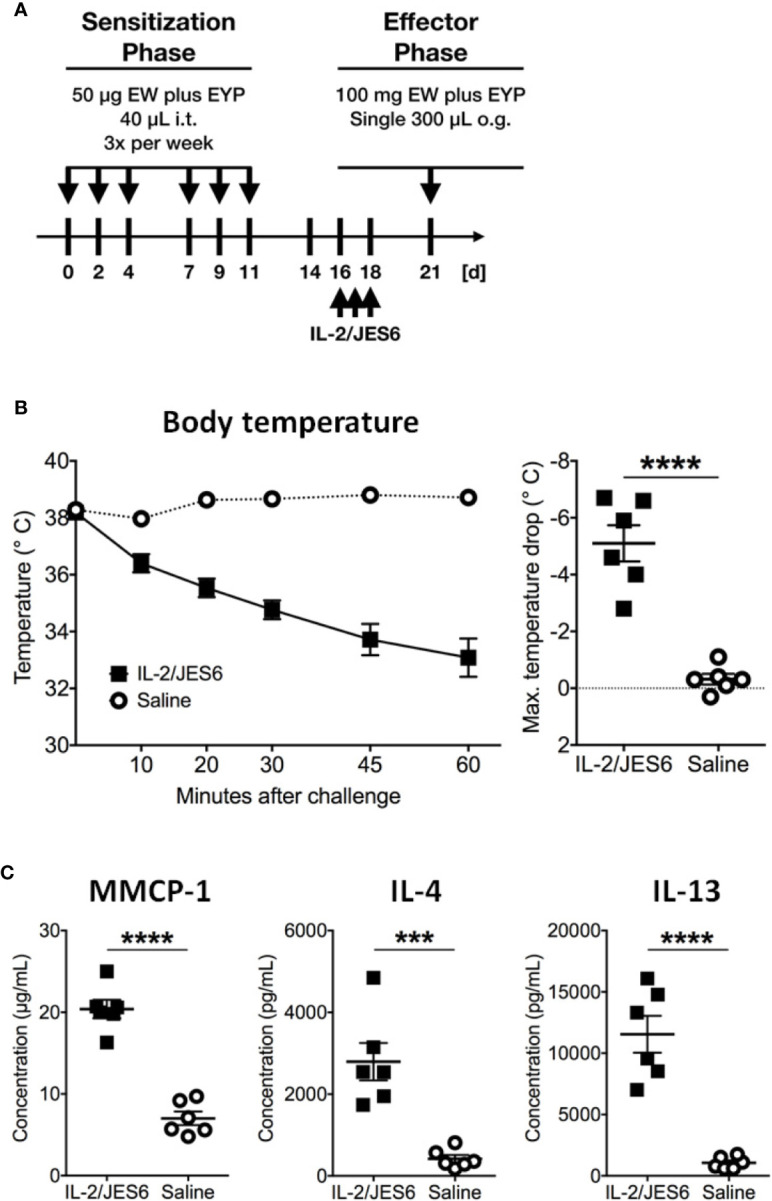
IL-2/JES6 treatment after allergen sensitization. Mice were treated with IL-2/JES6 after sensitization and before the first challenge. **(A)** Treatment regimen. **(B)** Rectal temperature curve (left) and maximal temperature drop (right) after antigen challenge. **(C)** Serum mMCP-1 as assessed by ELISA. IL-4 and IL-13 were measured by IVCCA. Data shown represent mean ± SEM (n = 6). ***p < 0.001, ****p < 0.0001 (Student’s t-test).

Mice challenged 72 h after IL 2/JES6 treatment still developed increased systemic anaphylaxis (**Figure 2C**), although less severe than mice challenged 4 h after IL 2/JES6 treatment. As indicated by increased MMCP-1 levels, IL 2/JES6 treatment led to an exacerbated mast cell response, while OVA-specific IgE levels were not affected (**Figure 2D**). In order to determine if allergen-sensitization alone is sufficient to induce susceptibility to IL-2/JES6-mediated exacerbation of anaphylaxis, mice were treated with IL 2/JES6 directly after allergen sensitization but before the first oral allergen challenge (**Figure 3A**). Although saline-treated mice developed neither hypothermia nor diarrhea to the first oral challenge, sensitized and IL 2/JES6-treated mice developed an approximately 5°C drop (**Figure 3B**). Consistently, compared to controls, IL 2/JES6-treated mice showed substantially increased MMCP 1, IL-4 and IL-13 levels in serum (**Figure 3C**).

**Figure 4:**
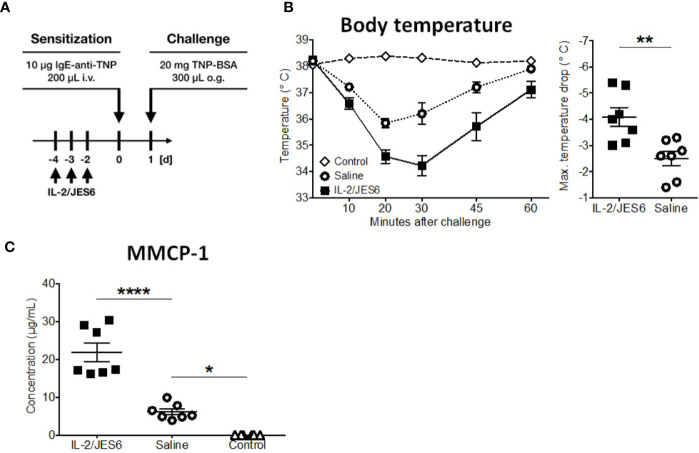
IL-2/JES6 treatment enhances mast cell activation and aggravates IgE-transfer-mediated systemic anaphylaxis. Mice were treated with IL-2/JES6 or saline, passively sensitized to trinitrophenyl (TNP) by transfer of monoclonal anti-TNP IgE and subsequently challenged with TNP-BSA. **(A)** Treatment regimen. **(B)** Rectal temperature curve (left) and maximal temperature drop (right) following oral challenge. **(C)** Serum mMCP-1 as assessed by ELISA. Data represent mean ± SEM (n = 6–7). Data shown from one of two independent experiments. **p < 0.01 (Student’s t-test); *p < 0.05, ****p < 0.0001 (one-way ANOVA followed by Tukey’s *post hoc* test).

Together, these experiments demonstrate that IL-2/JES6 administration that is first administered after mice have been sensitized does not affect allergen-specific IgE, but exacerbates anaphylaxis and increases the allergen-reactivity of IgE-sensitive effector cells, most likely mast cells. To investigate the role of Ag-specific IgE further, we used an IgE transfer model of systemic anaphylaxis that depends solely on mast cell activation, but circumvents the induction of an allergen-specific B and T cell response (3, 45). Mice were treated with three daily doses of IL-2/JES6. Two days after the last IL-2/JES6 dose they were passively sensitized with a single dose of monoclonal murine IgE-anti-trinitrophenyl (TNP) and 24 h later challenged with TNP-BSA (**Figure 4A**). IL-2/JES6 treatment increased the anaphylactic and MMCP-1 responses to allergen-challenge in mice that had received IgE-anti-TNP (**Figures 4B, C**). Thus, the IL-2/JES6-induced increase in mast cell degranulation and anaphylaxis severity is at least partially independent of increased Ag-specific IgE levels.

Collectively, these data indicate that the IL-2 agonist increases the allergen-responsiveness of mast cells in the presence of IgE, which under pathophysiological conditions is the case in already sensitized individuals.

### IFN-γ Mediates Exacerbation of Acute Anaphylaxis by IL-2/JES6

To determine how IL-2/JES6 increases IgE-mediated mast cell responses and exacerbates anaphylaxis, we investigated the effects of IL-2/JES6 treatment on the size of mast cell populations in the gut, early events in mast cell activation, and cytokine responses by T helper cells.

**Figure 5:**
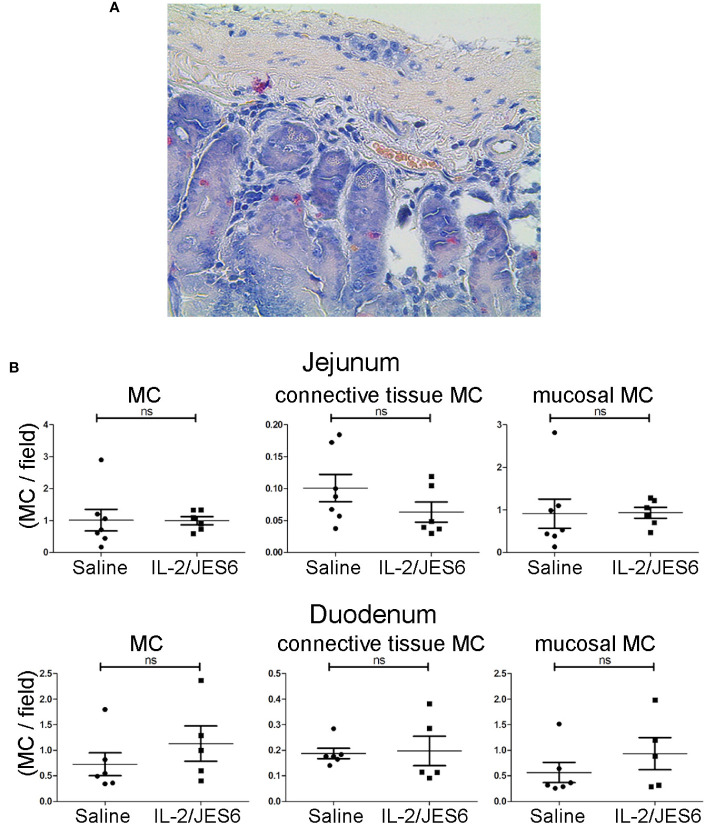
Mast cell populations in the gut. Gut sections from sensitized mice treated with IL-2/JES6 or PBS for three days prior to sensitization. Mast cells were identified by chloroacetate esterase (CAE) staining. **(A)** Representative section of duodenum, 40x magnification. **(B)** All mast cells, connective tissue mast cells and mucosal mast cells in jejunum (upper row) and duodenum (lower row) are shown, as indicated. Plots show mast cells per field of view (MC/field). Upper row: jejunum, lower row: duodenum, as indicated. Each dot represents the average mast cell counts from one individual mouse, two to six slices were counted per mouse (n = 5; Mann-Whitney U test). ns, not significant.

Allergic mice repeatedly treated with IL-2/JES6 did not have increased mast cell populations in either the mucosa or connective tissues of the jejunum and the duodenum (**Figure 5**); thus, IL-2 agonist neither promotes mast cell proliferation nor increases mast recruitment to the gut.

In order to test whether IL-2/JES6 has an immediate effect on mast cell activation, mice were sensitized to TNP by injection of IgE-anti-TNP and received a single injection of the IL-2 agonist 20 h later. Another 4 h later, the mice were challenged with TNP-BSA. After this single, short treatment, IL-2/JES6 did not promote anaphylaxis (**Figure S4**), suggesting that more prolonged treatment with the IL-2 agonist is required to affect mast cell responsiveness.

**Figure 6:**
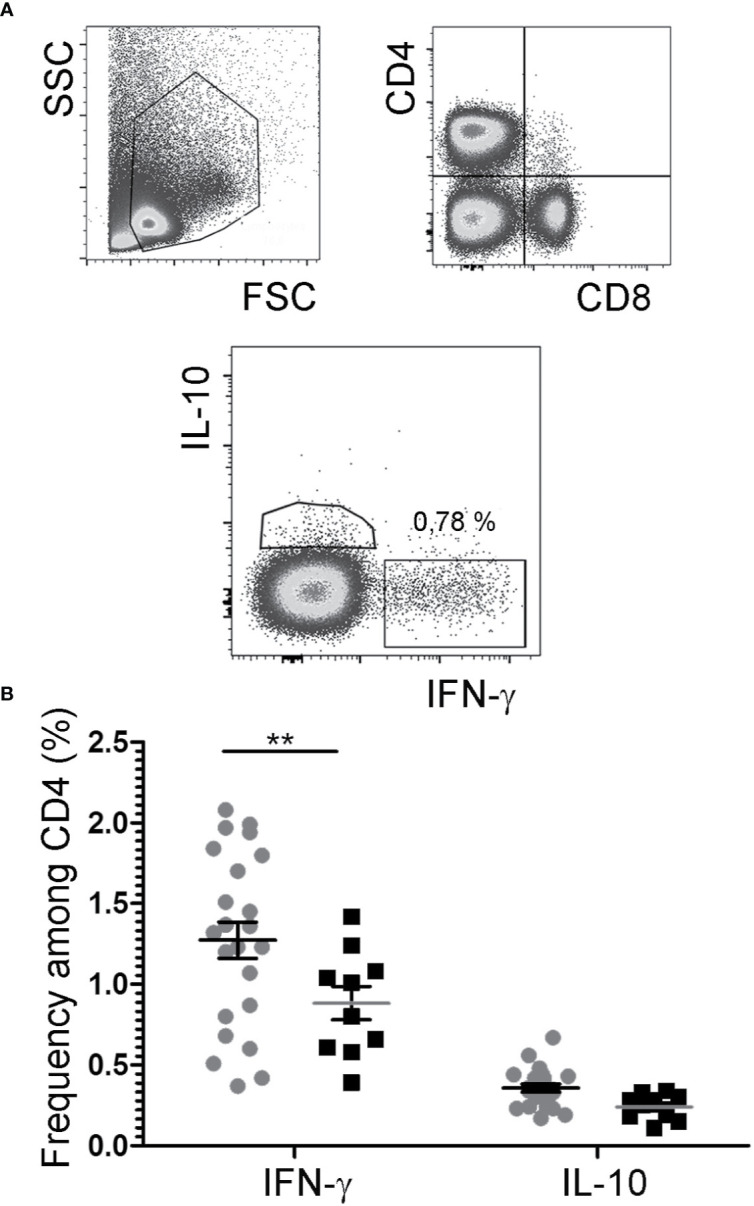
Cytokine expression by CD4+ T cells. After challenge 15, allergic mice were therapeutically inoculated for three days with IL-2/JES6 and analyzed 4 h after the last IL-2/JES6 injection. Cells from mesenteric LNs were stained for lineage markers and intracellular cytokine expression and analyzed by flow cytometry. **(A)** Gating. **(B)** Percentage of IFN-γ- and IL-10-expressing CD4+ cells in IL-2/JES6 (circles) and PBS treated controls (squares). Data represent mean ± SEM (n = 10 to 23). **p < 0.01 (two-way ANOVA with Bonferroni past *post hoc* test).

While IL-2/JES6 neither had immediate effects on mast cells, nor led to the expansion of mast cell populations, repeated applications of the IL-2 agonist increased the frequencies of IFN-γ+ cells within the CD4+ population, but had no impact on the frequencies of IL-4+/CD4+, IL-10+/CD4+, and IL-17A+/CD4+ cells (**Figure 6**; **Figure S5**).

**Figure 7:**
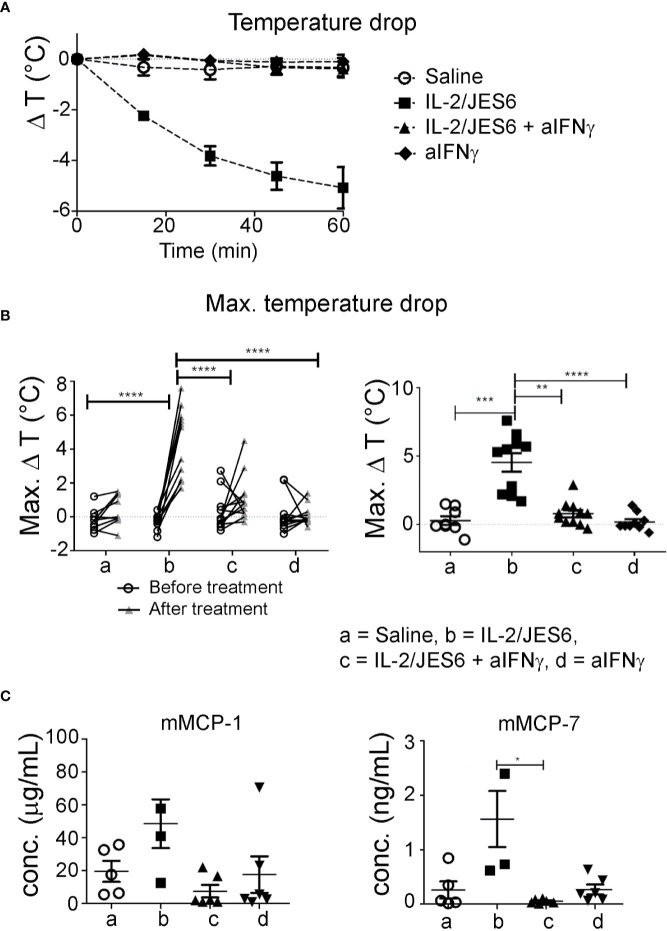
Impact of IFN-γ blockade. Egg-allergic mice were treated daily on three consecutive days with IL-2/JES6 and challenged with allergen after the last IL-2/JES6 injection. Some groups additionally received one or three injections of anti-IFN-γ blocking antibodies, with all or only the last injection of IL-2/JES6, respectively. Mice were challenged 4 h after the last IL-2/JES6 injection. **(A)** Temperature drop curve. Representative data from one of two experiments are shown **(B)** Changes of maximal temperature drops before and after anti-IFN-γ blockade in individual mice (left, statistics: two way ANOVA with Sidak’s multiple comparison test) and maximal temperature drops after treatment in the various groups (right, statistics: Kruskal-Wallis Test with Dunn’s multiple comparison test) are shown, as indicated. Data are pooled from two independent experiments. **(C)** Blood was drawn 1.5 h after the challenge and serum levels of mMCP-1 and mMCP-7 were measured by ELISA. Data represent mean ± SEM. Representative data from one of two experiments are shown (statistics: Kuskal-Wallis Test with Dunn’s multiple comparison test). *p < 0.05, **p < 0.005, ***p < 0.001, ****p < 0.0001.

Of note, although IFN-γ is the prototypic Th1 cytokine and is known to counteract type-1 hypersensitivity, it has been previously reported to promote mast cell effector functions and contribute to disease pathology in a mouse model of chronic asthma (46). Therefore, we tested whether the IL-2/JES6-induced increase in IFN-γ production could be responsible for the increased mast cell response and exacerbated anaphylaxis observed in allergic mice. Mice were sensitized to hen’s egg and challenged repeatedly with EW plus EYP until they developed considerable titers of allergen-specific IgE but did not yet have diarrhea or hypothermia following allergen challenge. Afterwards, the mice were divided into various groups showing comparable IgE titers. These groups were treated on three consecutive days with either IL-2/JES6 or saline as a control, and received one or three injections of anti- IFN-γ blocking or control mAb. The mice were challenged 4 h after the last injection of anti-IFN-γ blocking antibodies. As before, mice not treated with the IL-2 agonist did not develop detectable temperature drops. In contrast, all mice treated with the IL-2 agonist but without IFN-γ blockade consistently showed temperature drops of approximately 5°C (**Figures 7A, B**). IFN-γ blockade completely inhibited the IL-2/JES6-induced temperature drops. In addition, mice treated with IL 2/JES6 had increased serum levels of mast cell-derived proteases that were also reversed by anti-IFN-γ blocking antibodies (**Figure 7C**). The IFN-γ dependence of IL-2/JES6 exacerbation of anaphylaxis and mast cell degranulation probably reflects a direct effect of IFN-γ on mast cells, because the addition of IFN-γ to cell cultures of bone marrow-derived mast cells generated from wild-type mice, but not from IFN-γ receptor-deficient mice, has been shown to enhance mast cell responses *in vitro* (46).

Collectively, these results show that treating allergic mice with the IL-2 agonist IL 2/JES6 increases the IFN-γ response; which enhances IgE-mediated mast cell degranulation and consequently exacerbates allergic anaphylaxis.

## Discussion

Here, we established a novel model of food allergy to whole egg that involves sensitization through a biologically relevant route (the airways) without a requirement for an exogenous adjuvant. Using this model, we confirmed that IL-2 agonist treatment prior initial exposure to allergen, inhibits allergic sensitization and prevents allergen challenge-induced anaphylaxis. However, we also found that IL-2 agonist treatment drastically intensifies IgE-dependent mast cell responses and allergic anaphylaxis when administered to already sensitized mice and that this mechanism, surprisingly, depends on IFN-γ.

Designing experimental models of food allergy that resemble the pathophysiology of the human disease provides a challenge (47). Most murine egg allergy models use model antigens, such as OVA, which initially are applied together with artificial adjuvants. Intraperitoneal injection with alum adjuvant is the typical route for sensitization, although this has been replaced, in some studies, by epicutaneous sensitization through abraded skin. Intraperitoneal injection of OVA with alum is a strong, albeit artificial stimulus that allows the use of commercially available genetically modified mouse strains that express allergen-specific B and T cell receptors. However, chicken’s egg contains several allergens. While OVA is the most abundant EW protein, ovomucoid is the most allergenic (48). Furthermore, in addition to several protein allergens, hen’s egg contains high concentrations of saturated fat in egg yolk that appear to act as a natural adjuvant for the induction of food allergy, an effect that has been observed with saturated medium chain triglycerides (47) and U. Samavedam et al., manuscript in preparation. The combination of multiple protein allergens with lipid adjuvants in egg is likely to be important in the pathogenesis of food allergy to hen´s egg. In addition to mimicking the clinically relevant sensitization of humans with whole egg, rather than a single egg protein, our airway sensitization scheme has the advantage of mimicking a natural route of sensitization of humans to eggs, as established for patients who have developed the bird-to-egg syndrome (49, 50) or have inhaled powdered egg during its manufacture (17–20).

The most novel aspect of our study, however, is not the sensitization procedure used, but rather the observation that IL-2 exacerbates disease in sensitized individuals by inducing an IFN-γ response. Although the Th2 cytokine IL-4 is well known to play a critical role for the induction of immunoglobulin class switch to IgE and the development of food allergy (51–54), the prototypic Th1 cytokine IFN-γ can inhibit allergy by suppressing IL-4-induced IgE synthesis. Accordingly, shifting the immune response toward the Th1 phenotype can have beneficial effects on IgE-mediated allergic diseases (7, 55, 56).

In contrast, despite its protective role during the initiation of IgE-mediated allergies, IFN-γ has been shown to contribute to the pathogenesis of allergic asthma, particularly in patients with severe disease and during its chronic phase (46, 57–59). The development of full blown disease in one murine model of chronic asthma has been shown to depend on mast cell expression of IFN-γ receptor-1 (46). IFN-γ mediated signals seem to promote airway inflammation in this model in multiple ways, including increasing IgE-dependent mast cell histamine production.

Our data now demonstrate that IFN-γ can also exacerbate established food allergy. This effect similarly depends on allergen-specific IgE, appears to involve enhanced mast cell mediator (protease) production of mast cell proteases rather than an increase in mast cell number (46), takes > 1 day to develop and dissipates over a few days in the absence of elevated IFN-γ levels. Treatment with the IL-2 agonist IL 2/JES6 led to increased IFN-γ production by CD4 T cells, and IFN-γ was responsible for increased anaphylaxis, most likely through stimulation of mast cells. Because we show that the IL-2 agonist effect can occur in 3 days or less and IFN-γ -dependent and IL-2 predominantly induces rapid IFN-γ production by NK cells (60), it is likely that IL-2 induction of IFN-γ production by these cells is also involved.

In addition to the likelihood that IFN-γ directly enhances the mast cell response to FcεRI-mediated stimulation (46), it is possible that IFN-γ increases mast cell Ag presentation, which promotes mutually stimulatory mast cell-T cell interactions that could enhance effector molecule production, cytokine production, and mast cell degranulation by reducing the threshold for mast cells activation *via* FcγRI (61, 62). Consistent with this possibility, IFN-γ has been shown to increase mast cell MHC II expression (63, 64). Although IFN-γ can inhibit mast cell functioning and even kill mast cells in the absence of FcεRI crosslinking, FcεRI crosslinking reverses this inhibition and increases mast cell antigen presentation *via* MHCII (65, 66). This may well apply to our model, in which mast cells are already loaded with allergen-specific IgE when they encounter increased levels of IFN-γ and in which FcεRI crosslinking is induced by allergen-challenge.

Our observation that IL-2-induced IFN-γ can exacerbate food allergy may appear to contradict previous observations that endogenously produced IFN-γ inhibits allergy. One explanation for this apparent difference is that IL-2/JES6 treatment of sensitized mice increases their IL-4 and IL-13 responses to the sensitizing Ag as observed in our study, in addition to greatly increasing IL-2 levels, while the TLR agonists and herbal extract used in previous studies to increase IFN-γ production suppress the production of Th2 cytokines (67–70). Consequently, the presence of IL-2, IL-4 and/or IL-13 may unmask an enhancing effect of IFN-γ on mast cell responses, while IFN-γ in the absence of these cytokines has an inhibitory effect.

Our observation that IFN-γ exacerbates established food allergy has clinical implications. The efficacy of low-dose IL-2 therapy, which has been shown to restore Treg populations in systemic lupus erythematosus, is currently being evaluated in clinical trials. Our findings raise the concern that such therapy could be problematic in food-allergic patients. The impact of IFN-γ during established food allergy reveals also novel aspects on the relationship between Th2/IgE-mediated allergies and infections. IFN-γ is produced during the immune response against many infectious pathogens (71, 72). The hygiene hypothesis stipulates that a low prevalence of infection results in a Th2 bias and eventually contributes to the development of atopic disorders, including food allergies. However, epidemiological studies have failed to demonstrate a clear negative correlation between food allergy and infection. While some papers report that infection with Helicobacter pylori and Epstein-Barr virus increases the risk of developing food allergy (73–76), other studies found no correlation between Helicobacter pylori infection and food allergy or showed that this infection is associated with a decreased food allergy risk (77, 78). These inconsistent results are consistent with our observation that IFN-γ can inhibit food allergy during the sensitization stage, but exacerbate established food allergy.

## Data Availability Statement

The raw data supporting the conclusions of this article will be made available by the authors, without undue reservation.

## Ethics Statement

The animal study was reviewed and approved by local Committee on the Ethics of Animal Experiments of the state Schleswig-Holstein (Ministerium für Landwirtschaft, Umwelt und ländliche Räume des Landes Schleswig-Holstein). Animal studies performed in Cincinnati were approved by the Cincinnati Children’s Hospital Medical Center IACUC (IACUC2014-0041, expires December 12, 2020).

## Author Contributions

CL, CR, FF, CU, AB, and RM provided substantial contributions to the conception of the work. CL, CR, CU, MR, RK, SC, A-KC, TL, BF, KH, LA and AB performed the experiments. All authors contributed to the article and approved the submitted version.

## Funding

This work was supported by the international research training group “IRTG 1911.” AB, BF, and RM were supported by the Excellence Cluster “Inflammation at Interfaces,” and KH was supported by the graduate program “GRK 1727.” A-KC was funded by the Clinical Research Unit 303 “Pemphigoid Diseases—Molecular Pathways and their Therapeutic Potential” (CRU303). FF was supported by NIH grants R01 AI113162, R01AI145991 and R01AI130103.

## Conflict of Interest

The authors declare that the research was conducted in the absence of any commercial or financial relationships that could be construed as a potential conflict of interest.
