# Supplementary material for: IL-2-Agonist-Induced IFN-γ Exacerbates Systemic Anaphylaxis in Food Allergen-Sensitized Mice
Source: Front Immunol. 2020 Dec 11;11:596772. doi: 10.3389/fimmu.2020.596772 (PMC7759672; doi:10.3389/fimmu.2020.596772)
Supplement: Supplementary file 1 [file DataSheet_1.pdf]

Supplementary Material

1.1 Supplementary Figures

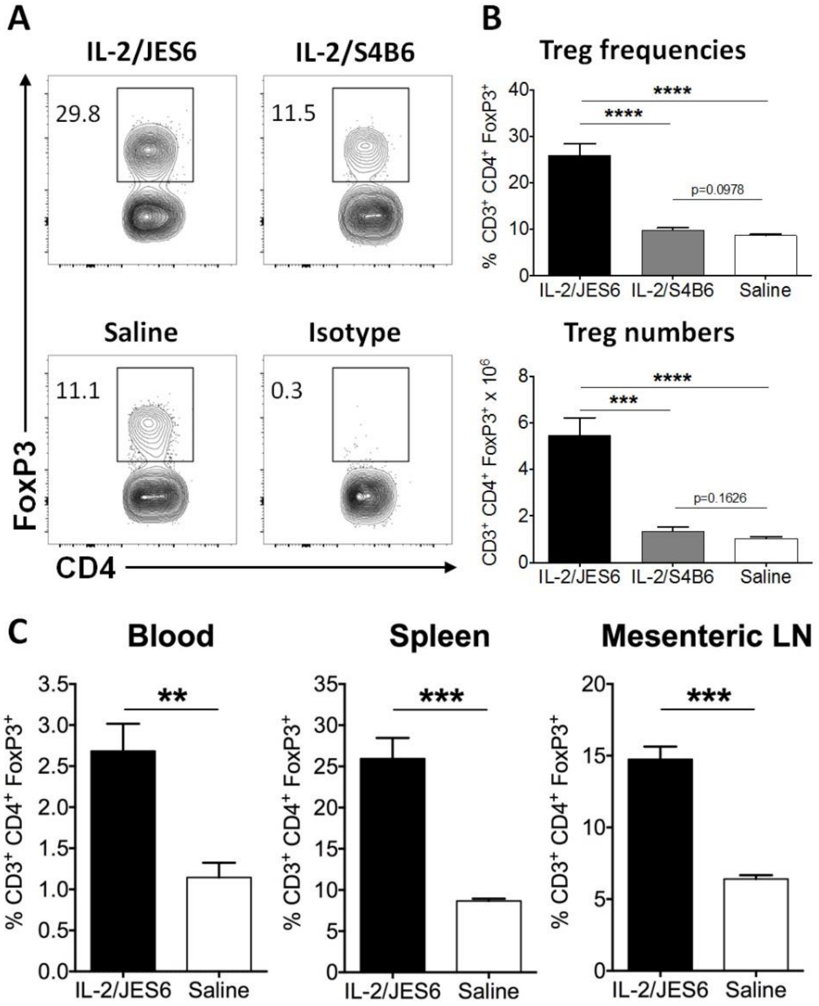

Supplementary Figure 1. IL-2/JES6 mediates expansion of Treg populations in various organs. (A) Mice were i.p. injected with IL-2/JES6 or IL-2/S4B6 or saline on three consecutive days. 72 h after the final injection, CD3+ CD4+ FoxP3+ Tregs in spleens of treated mice were analyzed by flow cytometry. (B) Frequencies (upper bar graph) and numbers (lower bar graph) of Tregs in spleens of IL-2/JES6-, IL-2/S4B6- and saline-treated mice. (C) The systemic effect of IL-2/JES6 on Treg populations was determined for blood, spleen and mesenteric LN. Data shown are from one of two independent experiments (n = 3-4). Data depicted represent mean ± SEM. ***p<0.001, ****p<0.0001 (one-way ANOVA followed by Tukey's post hoc test). Data shown in C represent mean ± SEM of 5-6 mice per group analyzed in one experiment. **p<0.01, ***p<0.001 (Student's t-test).

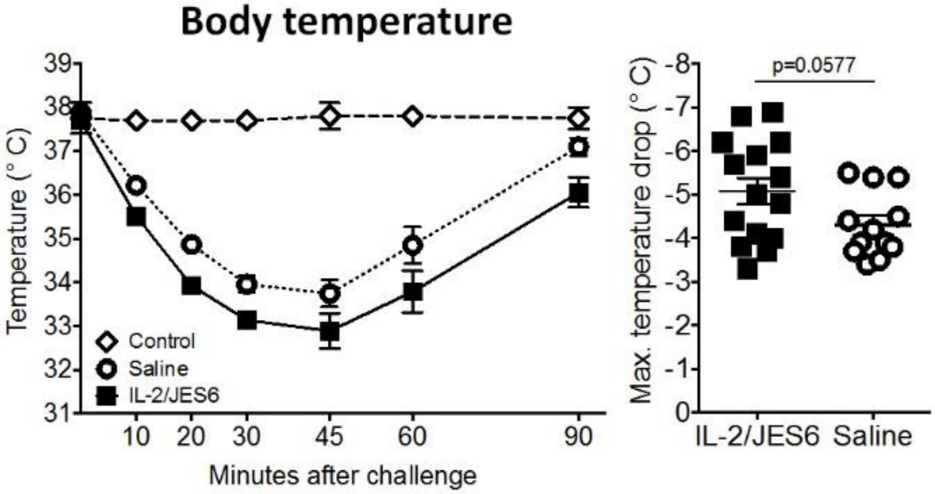

Supplementary Figure 2. Mice prophylactically treated with IL-2/JES6 are sensitive to histamine. Naïve mice were prophylactically treated with IL 2/JES6 or saline. Mice were then sensitized to EW plus EYP and subsequently challenged 8 times by o.g. Two days later, mice were i.v. injected with 0.75 mg histamine. Three mice of the saline-treated group (control) were injected with saline instead of histamine. Rectal temperature curve (left) and maximal temperature drop (right) following histamine challenge. Data shown represent mean ± SEM analyzed (Student's t-Test, n = 11-15)).

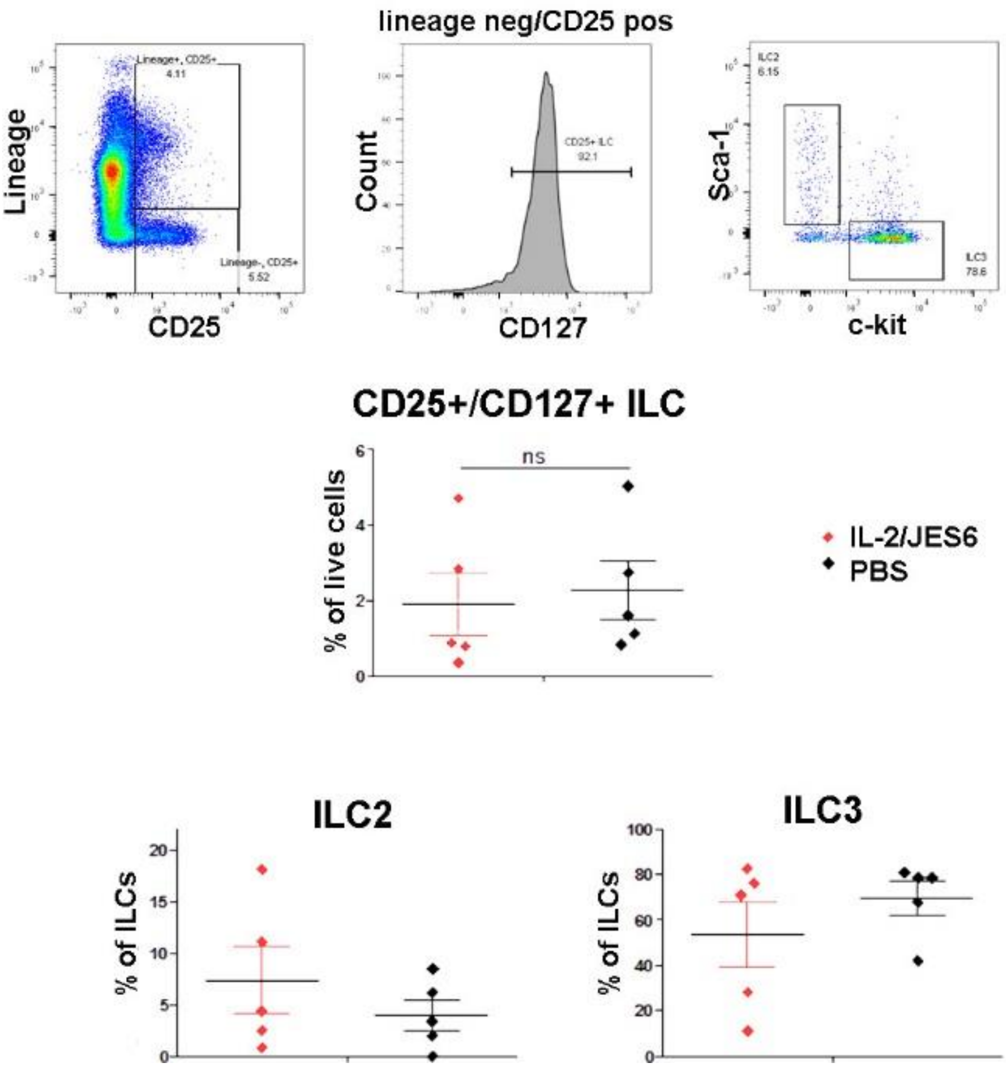

Supplementary Figure 3. Intestinal ILC populations. ILC Balb/c mice were sensitized to and challenged 15x with EW/EYP. IL-2/JES6 or PBS was injected 3x with the last injection 4 h prior to the final challenge. After the challenge, lamina propria lymphocytes were isolated and stained for flow cytometrical analysis. In order to identify LC, cells expressing the lineage markers CD3, B220, CD11b, Gr-1, Ter-119, CD49b, CD326 were excluded and LC were identified by expression of CD25 together with CD127. ILCs were further differentiated into ILC2 (c-kit negative, Sca-1 positive) and ILC3 (c-kit positive, Sca-1 negative). Histograms show the percentages of total ILC, ILC2 and ILC3, as indicated. Statistics: n=5 per group; ns: $p > 0.05$; Mann-Whitney U test

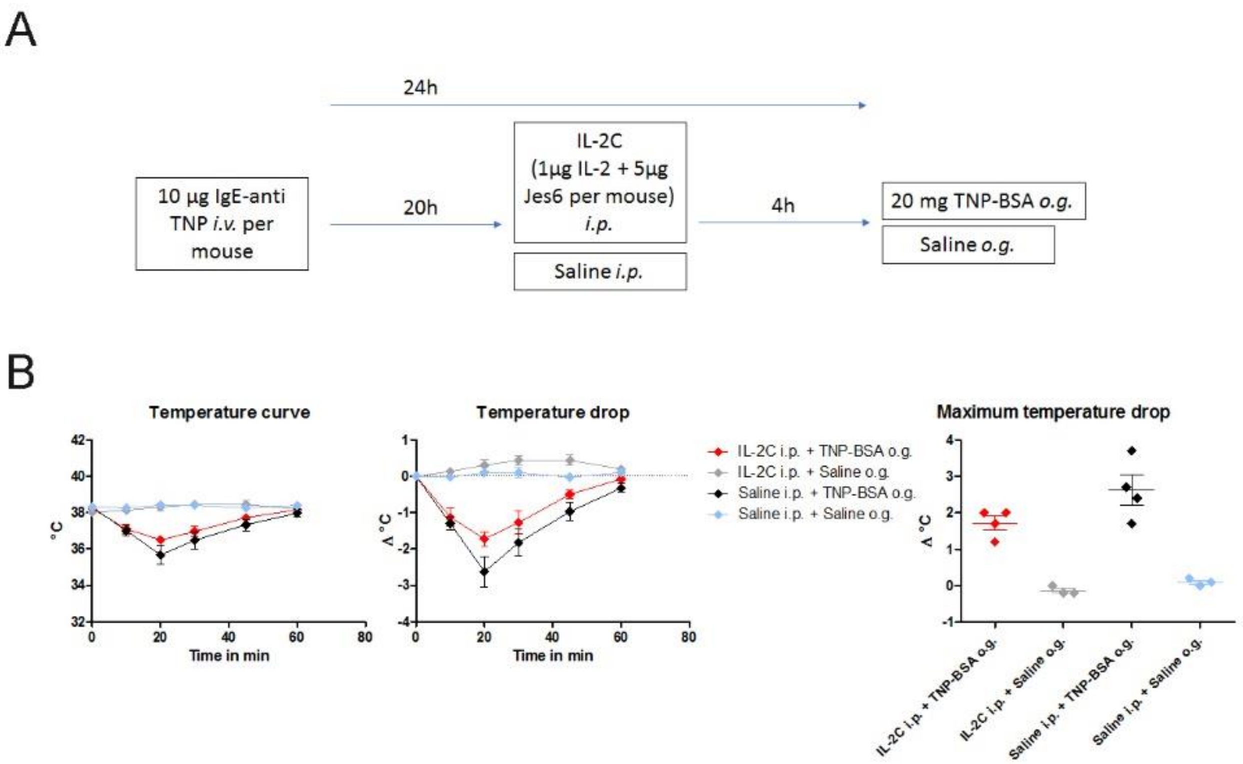

Supplementary Figure 4. IL-2/JES6 treatment shortly before allergen-challenge has no impact on IgE-transfer mediated systemic anaphylaxis. (A) Treatment regimen: Mice were treated with monoclonal anti-TNP IgE or saline as a control. After 20 hours, mice received IL-2/JES6 (IL-2 complex = IL-2C) or saline. Another four hours later mice were challenged. (B) Rectal temperature curve (left), temperature drop (middle) and maximal temperature drop (right) following oral TNP-BSA challenge, as indicated. Data represent mean ± SEM. No significant differences were determined by one-way ANOVA followed by Tukey’s post hoc test (n = 4).

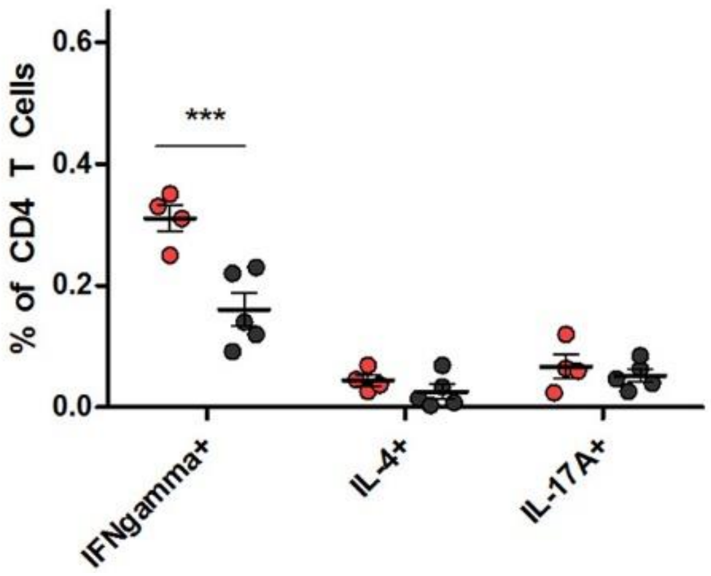

Supplementary Figure 5. Cytokine expression by CD4 T cells. After challenge 15, allergic mice were therapeutically treated for 3 days with IL-2/JES6 and analyzed 4 hours after the last IL-2/JES6 injection. Cells from mesenteric LNs were stained for lineage markers and intracellular cytokine expression and analyzed by flow cytometry. Shown are the percentages of IFN-γ, IL-4, and IL-17A expressing CD4+ cells in IL-2/JES6 (red) and PBS treated controls (grey). Data represent mean ± SEM (n = 4 to 5). ***$p<0.001$, (two-way ANOVA Bonferroni post hoc test).
